# An Archaeal Homolog of Proteasome Assembly Factor Functions as a Proteasome Activator

**DOI:** 10.1371/journal.pone.0060294

**Published:** 2013-03-21

**Authors:** Kentaro Kumoi, Tadashi Satoh, Kazuyoshi Murata, Takeshi Hiromoto, Tsunehiro Mizushima, Yukiko Kamiya, Masanori Noda, Susumu Uchiyama, Hirokazu Yagi, Koichi Kato

**Affiliations:** 1 Graduate School of Pharmaceutical Sciences, Nagoya City University, 3-1 Tanabe-dori, Mizuho-ku Nagoya, Japan; 2 National Institute for Physiological Science, National Institutes of Natural Sciences, 5–1 Higashiyama, Myodaiji, Okazaki, Aichi, Japan; 3 Picobiology Institute, Department of Life Science, Graduate School of Life Science, University of Hyogo, 3-2-1 Kouto, Kamigori-cho, Ako-gun, Hyogo, Japan; 4 Okazaki Institute for Integrative Bioscience and Institute for Molecular Science, National Institutes of Natural Sciences, 5-1 Higashiyama, Myodaiji, Okazaki, Aichi, Japan; 5 Department of Biotechnology, Graduate School of Engineering, Osaka University, 2-1 Yamadaoka, Suita, Osaka, Japan; University of Florida, United States of America

## Abstract

Assembly of the eukaryotic 20S proteasome is an ordered process involving several proteins operating as proteasome assembly factors including PAC1-PAC2 but archaeal 20S proteasome subunits can spontaneously assemble into an active cylindrical architecture. Recent bioinformatic analysis identified archaeal PAC1-PAC2 homologs PbaA and PbaB. However, it remains unclear whether such assembly factor-like proteins play an indispensable role in orchestration of proteasome subunits in archaea. We revealed that PbaB forms a homotetramer and exerts a dual function as an ATP-independent proteasome activator and a molecular chaperone through its tentacle-like C-terminal segments. Our findings provide insights into molecular evolution relationships between proteasome activators and assembly factors.

## Introduction

The proteasome is a major proteolytic machine for selective protein degradation in cells. It forms huge protein complexes consisting of a catalytic core particle (20S proteasome) and one or two proteasome activators [1], [2], [3]. The 20S proteasome is composed of seven α subunits and seven β subunits and forms a cylindrical architecture of four heptameric rings with two outer α subunit rings and two inner β subunit rings [4], [5]. The 20S proteasome avoids nonselective proteolysis by virtue of its architecture, which sequesters its proteolytic sites in the central chamber of the cavity. Substrate entry is restricted by a gating pore at the center of the α subunit ring, which opens when this ring interacts with proteasome activators.

The 19S regulatory particle (also called PA700), PA26/PA28, and PA200/Blm10 function as proteasome activators in eukaryotic cells [6]. The 19S regulatory particle is composed of at least 19 subunits and can be divided into two subcomplexes, the base and the lid, whereas PA26/PA28 and PA200/Blm10 have homo/heteroheptameric and monomeric structures, respectively [7], [8]. The base of the 19S regulatory particle adopts a heterohexameric ring constructed by six different AAA-ATPase subunits (Rpt1–Rpt6) [1], [2], [3], [6]. These open the gate of the 20S proteasome and unfold and subsequently translocate the substrates therein in an ATP-dependent manner [3], [6]. Conversely, PA26/PA28 and PA200/Blm10 stimulate the degradation of peptides but not folded proteins in an ATP-independent manner [9], [10]. In archaea, while proteasome-activating nucleotidase (PAN), an ortholog of Rpt AAA-ATPases, has been identified [11], [12], an ATP-independent proteasome-activating mechanism has yet to be reported [13]. Gate-opening mechanisms among eukaryotic and archaeal proteasome activators are essentially identical. They insert the C-terminal peptide segment into a pocket formed between adjacent α subunits, thereby stimulating induced-fit conformational changes that lead to gate opening [7], [8], [14], [15], [16]. Unlike PAN/PA700 and PA200/Blm10, PA26/PA28 lacks a penultimate tyrosine residue at the C-terminus and, instead, uses an internal activation loop to open the gate [7]. Some archaeal species (e.g., *Thermoplasma* and *Pyrobaculum* spp.) lack the gene encoding PAN; therefore, the existence of other proteasome activator(s) in archaea has been postulated [13]. Very recently, it has been reported that AAA+-ATPase Cdc48 from *Thermoplasma acidophilum* functions as an ATP-dependent proteasome activator [17].

The molecular mechanisms underlying proteasome assembly have been extensively investigated in recent years. The accumulated evidence indicates that the assembly of the eukaryotic 20S proteasome is not spontaneous self-organization but a highly ordered process assisted by several proteins that transiently interact with proteasome assembly intermediates [18], [19]. Formation of the 20S proteasome is assisted by the assembly factors UMP1 and PAC1 – PAC4. Conversely, archaeal 20S proteasomes are typically composed of seven identical α subunits and seven identical β subunits, which spontaneously assemble into four-stacked homoheptameric rings *in vitro* [4]. Intriguingly, recent bioinformatic analysis identified the archaeal PAC1-PAC2 homologs PbaA and PbaB from *Methanococcus maripaludis* [20]. The homologs of these proteins are widely distributed among archaea, often with a C-terminal, proteasome-binding HbYX motif that has been found in several proteasome activators including Rpt ATPase and PA200/Blm10 and the proteasome assembly factor PAC1 [6]. It has been reported that the *M. maripaludis* PbaA preferentially binds a 20S proteasome precursor that retained β subunit propeptides through its HbYX motifs [20]. However, it remains unclear how the Pba proteins contribute to proteasome assembly in archaea, which can proceed spontaneously *in vitro* and presumably also in cells.

To elucidate the functional role of assembly factor-like proteins in archaea, we conducted biochemical and structural studies of *Pyrococcus furiosus* Pba proteins. We discovered that the archaeal PAC1-PAC2 homolog PbaB functions as an ATP-independent proteasome activator.

## Results and Discussion

### PbaB binds the mature 20S proteasome

**Figure 1:**
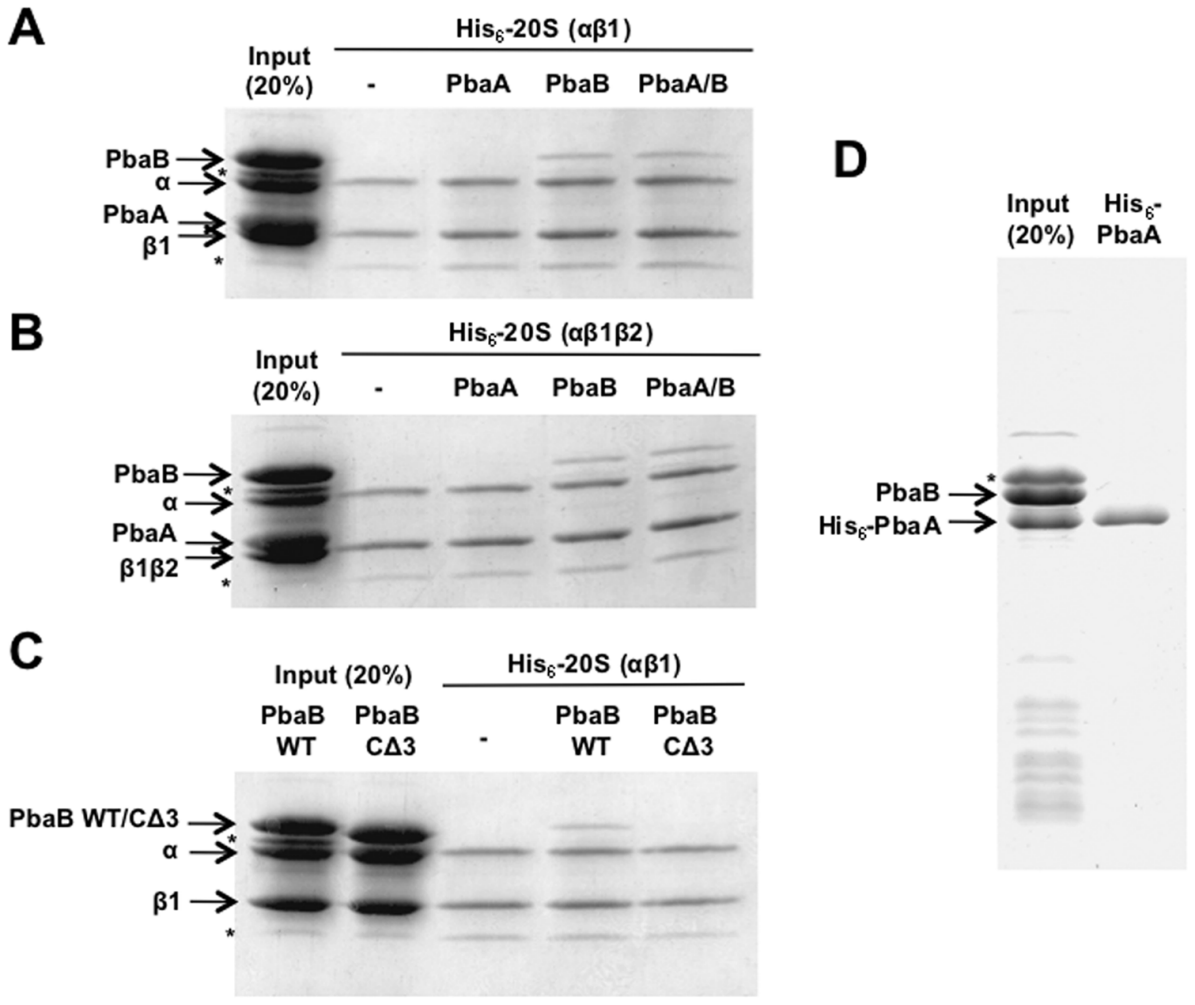
PbaB binds the mature 20S proteasome through its HbYX motif. (A and B) PbaB binds to the mature 20S proteasomes (A) αβ1 form and (B) αβ1β2 form but PbaA does not. The non-tagged PbaA and/or PbaB were mixed with the mature 20S proteasome [His_6_-tagged inactive mutants shown as His_6_-20S(αβ1) and His_6_-20S(αβ1β2)] immobilized on Ni^2+^-charged chelating Sepharose beads. After extensive washing, bound proteins were analyzed by CBB staining after SDS-PAGE. (C) PbaB binds to the mature 20S proteasome (αβ1 form) through its HbYX motif. The PbaB mutant lacking the three amino acid residues constituting the HbYX motif (CΔ3) did not interact with the mature 20S proteasome. (D) PbaB does not form a heteromeric complex with PbaA. Pull-down experiments were performed using a lysate from the co-expressing cells. Asterisks indicate impurities.

We first characterized the possible interactions of PbaA and PbaB with the mature forms of 20S proteasomes from *P. furiosus* with catalytically inactive mutations. This organism has a single isoform of proteasome α subunit (PsmA) and two isoforms of proteasome β subunit (PsmB1 and PsmB2). Since β1 subunit has been reported to be indispensable for reconstruction of recombinant 20S proteasomes [21], we prepared two forms of 20S proteasomes composed of α subunit and hexahistidine (His_6_)-tagged β1 subunit with and without β2 subunit (termed as αβ1β2 and αβ1, respectively) for the interaction assays. Pull-down experiments showed that PbaB bound to both αβ1 and αβ1β2 proteasomes while binding of PbaA to the mature 20S proteasomes (αβ1 and αβ1β2) could not be detected, although it had an HbYX motif (Figure 1A and 1B). We revealed that binding of PbaB to the 20S proteasome depended on its C-terminal HbYX motif (Figure 1C). Even with a PbaA – PbaB mixture, only PbaB was detected in the proteasome-bound fraction. Co-expression data also demonstrated that these two Pba proteins did not form a heteromeric complex (Figure 1D). Instead, *P. furiosus* PbaA has been reported to form a complex with a hypothetical protein (PF0014) [22]. Since the binding level was almost identical between the αβ1 and αβ1β2 forms of proteasomes, we used αβ1 20S proteasome in the following experiments.

To address the possibility that PbaB binds a 20S proteasome precursor, we compared the affinities of PbaB for the mature 20S proteasome with those for its assembly intermediates in a quantitative manner. We prepared a non-tagged double homoheptameric ring of the α subunit and an immature 20S proteasome retaining the β subunit propeptide with an inactivating mutation at the active site (α + β1^T7A^) [20], [23]. The His_6_-PbaB was immobilized on the biosensor chip, and the proteasome and its intermediate mimics were injected as analytes. The dissociation constants of the α subunit ring and the immature or mature 20S proteasome were estimated to be 3.3±0.6×10^−7^ M and 2.1±0.3×10^−8^ M or 7.8±0.6×10^−9^ M, respectively (Figure S1). We also confirmed that the interaction between PbaB and the mature 20S proteasome depended on its C-terminal HbYX motif (Figure S1D). These data demonstrated that PbaB bound preferentially to the mature 20S proteasome rather than the proteasome assembly intermediates. This finding implied that the primary functional role of this protein may not be assistance of proteasome assembly.

### PbaB functions as a proteasome activator

**Figure 2:**
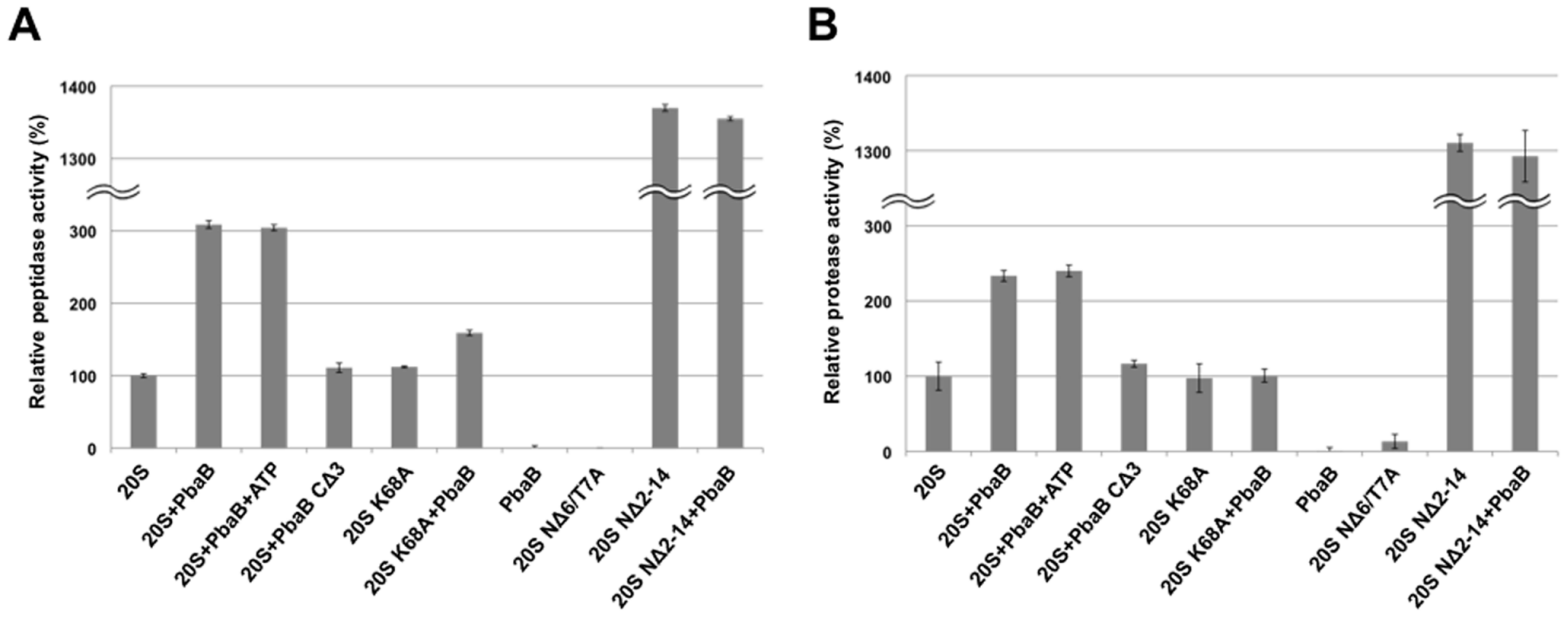
PbaB functions as a proteasome activator through the HbYX motif. PbaB accelerated proteasomal degradation of (A) fluorogenic nonapeptide substrate (LFP) and (B) α-synuclein in an HbYX motif-dependent manner. Mixture of the wild-type 20S proteasome and LFP or α-synuclein was incubated in the presence and absence of PbaB. The proteasomal activation by PbaB was independent of 1 mM ATP and was compromised by a K68A mutation. Gate-less mutant (NΔ2-14 proteasome α subunit from *P. furiosus*) had greater activities for LFP or α-synuclein hydrolysis, which was not further activated by PbaB. Error bars represent s.e.m. (n = 3).

The C-terminal HbYX motifs have often been found in proteasome activators including PAN [6]. Hence we ascertained whether PbaB could also activate the proteasome through its HbYX motif. As model substrates for probing possible proteasomal activation, we used a fluorogenic nonapeptide substrate [LFP, mca-AKVYPYPME-dap(dnp)-amide] and human α-synuclein, a natively unfolded protein. Intriguingly, PbaB accelerated proteasomal degradation of LFP and α-synuclein by about 3- and 2.5-fold, respectively (Figure 2), whereas the mutated PbaB that lacked the C-terminal HbYX motif (Gly-Tyr-Leu) did not accelerate proteasomal degradation. The stimulation activities of PbaB were comparable with those reported for PAN (3.4-fold and 4.3-fold for LFP and β-^14^C-casein, respectively) [24]. We confirmed that substrate degradation was not accelerated by PbaB alone (Figure 2) and no autodegradation was detected for the 20S proteasome and PbaB (Figure S2). These data indicated that the association of PbaB with the 20S proteasome through its HbYX motif, which is supposed to act as an α subunit ring gate opener [14], [15], [16], are prerequisite for proteasomal activation. Supporting this claim, we demonstrated that an alanine substitution of Lys68, which is involved in docking of the C-terminal HbXY motif of PAN [15], resulted in a significant impairment in the PbaB-dependent activation of the 20S proteasome. Furthermore, we showed that PbaB did not enhanced the proteolytic activity of the gate-less mutant (NΔ2-14 proteasome α subunit from *P. furiosus*), which already had greater activities for LFP and α-synuclein hydrolysis by 14-15 fold as compared with those of the wild-type 20S proteasome (Figure 2). Moreover, we revealed that PbaB activates the proteasome irrespective of the presence of ATP (Figure 2). Thus, PbaB was shown to be the first example of an archaeal proteasome activator that does not require ATP for acceleration of proteasomal degradation, whereas PAN facilitates unfolding of the proteasome substrate in an ATP-dependent manner. It has been reported that REGγ/PA28γ, an eukaryotic ATP-independent proteasome-activator, facilitates proteasomal degradation of not only small peptides but also specific protein substrates having unstructured regions such as steroid receptor co-activator SRC-3 and cyclin-dependent kinase inhibitor p21^Waf/Cip^1 [25], [26]. Since the 20S proteasome has an endoproteolytic activity against natively unfolded proteins [27], it has been suggested that REGγ proteasome-substrate complex targets the unstructured segments of these protein substrates [25], [26]. By analogy, we hypothesize that PbaB also contribute to proteasomal degradation of some specific protein substrates with unstructured region.

### PbaB homotetramer caps the 20S proteasome

To understand the molecular mechanisms of proteasome activation and substrate recognition by PbaB, we conducted structural biology analyses by electron tomography in conjunction with X-ray crystallography. Using the Au^3+^-bound crystal belonging to space group *C*222_1_ with four molecules per asymmetric unit, the atomic coordinates were determined by the single-wavelength anomalous dispersion (SAD) method. The final model of PbaB refined to a resolution of 2.20 Å has an *R*_work_ of 19.6% and *R*_free_ of 23.7% (Table 1), although the C-terminal 19, 21, or 23 residues including the HbYX motif gave no interpretable electron density. The crystal structure of PbaB exhibited a tetrameric structure with a remarkably large (1227 Å^2^) surface area buried through quaternary structure formation. Analytical ultracentrifugation also confirmed that PbaB forms a stable homotetramer in solution (Figure S3). These results were in marked contrast to those of their eukaryotic homologs PAC1 and PAC2, which have been reported to form a heterodimer [28].

**Table 1:** Data Collection and Refinement Statistics for PbaB.

	Native	NaAuCl_4_
**Crystallographic data**		
Space group	*C*222_1_	*C*222_1_
Unit cell *a*/*b*/*c* (Å)	115.6/156.4/153.0	115.8/156.8/153.9
**Data processing statistics**		
Beam line	SPring-8 BL44XU	SPring-8 BL44XU
Wavelength (Å)	0.90000	1.03514
Resolution (Å)	50–2.20 (2.24–2.20)	50–2.60 (2.64–2.60)
Total/unique reflections	504,879/69,848	248,821/43,044
Completeness (%)	99.7 (100.0)	99.6 (99.4)
* R*_merge_ (%)	6.8 (35.3)	7.3 (37.9)
* I*/σ (*I*)	50.3 (8.2)	33.8 (5.2)
Redundancy	7.3	5.8
**Refinement statistics**		
Resolution (Å)	20.0–2.20	
* R*_work_/*R*_free_ (%)	19.6/23.7	
R.m.s. deviations from ideal		
Bond lengths (Å)	0.018	
Bond angles (°)	1.57	
Ramachandran plot (%)		
Favored	92.9	
Allowed	7.1	

**Figure 3:**
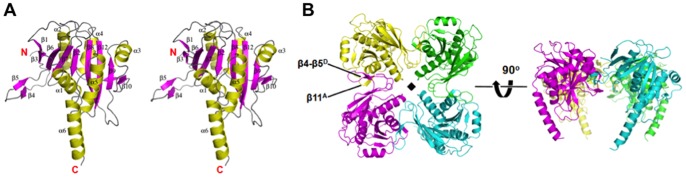
PbaB forms a homotetramer exhibiting tentacle-like C-terminal segments. (A) Stereo ribbon model of the PbaB monomer. β-strands, α-helices, and loops are shown in magenta, yellow, and gray, respectively. (B) Ribbon models of the PbaB tetramer are shown on the left and right. The two structures are related by a rotation of 90° around a horizontal axis. Chains A, B, C, and D are colored yellow, green, cyan, and magenta, respectively. The β-hairpin (β4–β5) makes a three-stranded anti-parallel β-sheet with β11 from the adjoining molecule (β11 of chain A and β4–β5 of chain D are highlighted). The non-crystallographic vertical fourfold axis is shown as a black square.

Each PbaB protomer has a single domain comprised of a central mixed β-sheet (β1–β3–β6–β7–β2–β8–β12–β9–β10) flanked by four α-helices (α1, α3, α5, and α6) on one side and one β-strand (β11) and two α-helices (α2 and α4) on the other side, thereby making an overall three-layered αβα fold (Figure 3A). Extended β-hairpin (β4–β5) is involved in tetramerization by forming a three-stranded anti-parallel β-sheet with β11 from the adjoining molecule (Figure 3B). In the tetramer structure, the C-terminal α-helices (α6) are located peripheral to the core domain and extend in the same direction, yielding a tentacle-like structure. The α6 helices showed poor electron densities with high crystallographic *B* values (45–100 Å^2^), suggesting that the C-terminal segments had flexible properties.

Comparative structural analysis using the program Dali server [29] identified uncharacterized protein Ta1441 from the archaeon *Thermoplasma acidophilum* (PDB: 3GAA) as a structural homolog (Figure S4). This protein shares 21.3% and 19.6% identities with *P. furiosus* PbaA and PbaB, respectively. It also possesses a C-terminal HbYX motif and forms a homopentamer in the crystal (Figure S4D). Structural alignment analysis between the Ta1441 and PbaB protomers revealed that their structures were essentially identical except that the β-hairpin involved in oligomerization was significantly deformed and the C-terminal α-helix was stabilized in contact with the core, forming a flat surface in Ta1441 (Figure S4). The amino acid sequences of these segments of Ta1441 are much closer to those of *P. furiosus* PbaA than of PbaB. Our analytical ultracentrifugation studies showed that PbaA formed a pentamer in solution (Figure S3). This finding suggested that the structural variation in the region corresponding to β4 and β5 of *P. furiosus* PbaB was a determining factor of the oligomeric states of the Pba homologs. In Ta1441 (and presumably in PbaA), the C-terminal segments are packed against the core. This may explain the inability of PbaA to bind to the 20S proteasome. *P. furiosus* PbaA interacts with a hypothetical protein (PF0014) [22]. It is possible that the PbaA pentamer alone is in a resting state but may become activated to enable proteasome binding after forming a complex with this protein. In contrast, in the PbaB tetramer, the C-terminal HbYX motifs inherently project probably toward the heptameric α-rings of the 20S proteasome.

**Figure 4:**
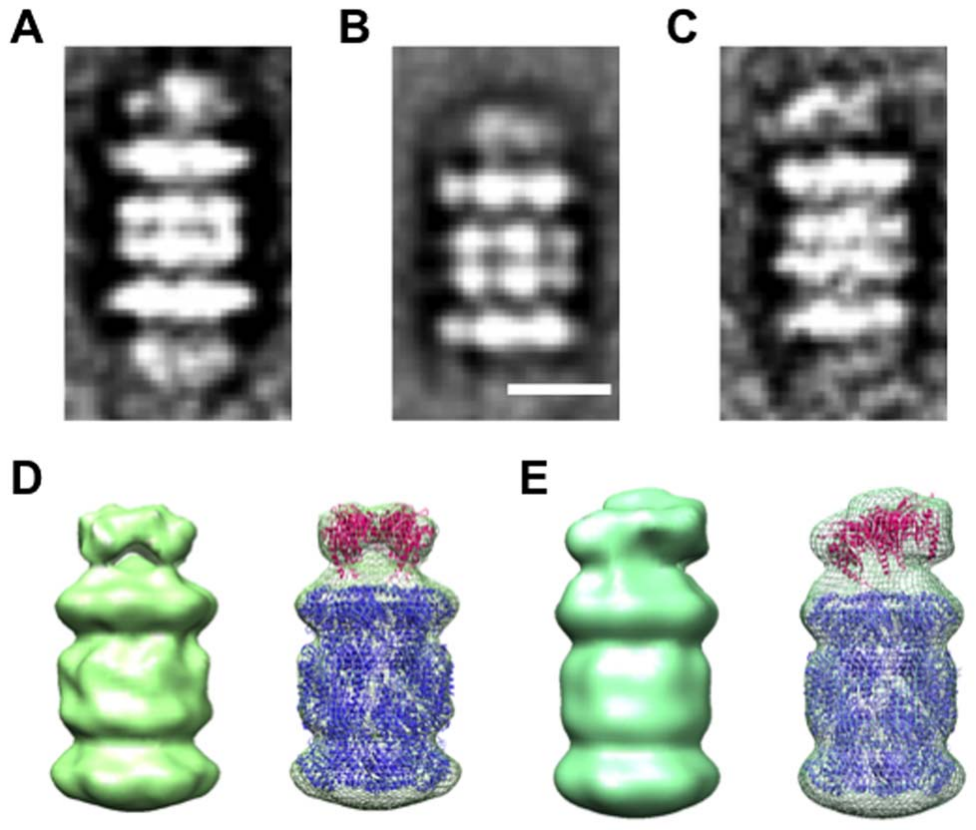
PbaB tetramer caps the heptameric α subunit ring of the 20S proteasome. Class averages of the PbaB-20S proteasome complexes; (A) the 20S proteasome doubly capped with PbaB on both sides, (B) and (C) the 20S proteasome singly capped with PbaB on one side. Scale bar indicates 10 nm. Three-dimensional tomograms for the PbaB-20S proteasome complexes were obtained by the negative staining electron tomography; (D) parallel cap (n = 9), (E) tilted cap (n = 42). Seven-fold symmetrical operation is applied to the 20S proteasome part.

To visualize the interaction mode between the PbaB and the 20S proteasome, we collected electron tomography images of the complexes, which were averaged after alignment and classification (Figure 4). In the total subtomograms (146 particles), the 20S proteasomes showed singly (103 particles) or doubly (36 particles) capped complexes with the PbaB tetramer on the α subunit ring. Fraction of the two forms of the complexes varied depending on the mixture ratio of PbaB and 20S proteasome. The crystal structures of the PbaB tetramer and the 20S proteasome (PDB ID:3MI0) well fit into the subtomogram averaged maps, respectively (Figure 4D and 4E). The three-dimensional structural features of the capped complexes is consistent with the observation that PbaB functions as a proteasome activator. Interestingly, among the singly capped complexes, 88.3% of the PbaB tetramer was significantly tilted with respect to the surface of the α subunit ring (Figure 4C and 4E), while 11.7% of the PbaB tetramer was in parallel to the surface of the α subunit ring fully capping it. There was no other option in terms of the interaction mode in our observation. The similar tendency was observed in the doubly capped complexes. The fractions of the complexes are summarized in Table 2. These results suggest that, in the major forms of the complexes, the PbaB tetramer partially caps the α subunit ring and a subset of C-terminal segments is not directly involved in the interaction with the 20S proteasome possibly due to a symmetry mismatch between the PbaB homotetramer and the α subunit homoheptameric ring.

**Table 2:** Structural fraction of the PbaB-20S proteasome complexes.

Singly capped 103 (70.5%)		Doubly capped 36 (24.7%)		Unbound 7 (4.8%)
Parallel cap	Tilted cap	Parallel cap	Tilted cap	-
12 (11.7%)	91 (88.3%)	7 (19.4%)	29 (80.6%)	-

### PbaB exerts chaperone activity through the tentacle-like C-terminal segments

**Figure 5:**
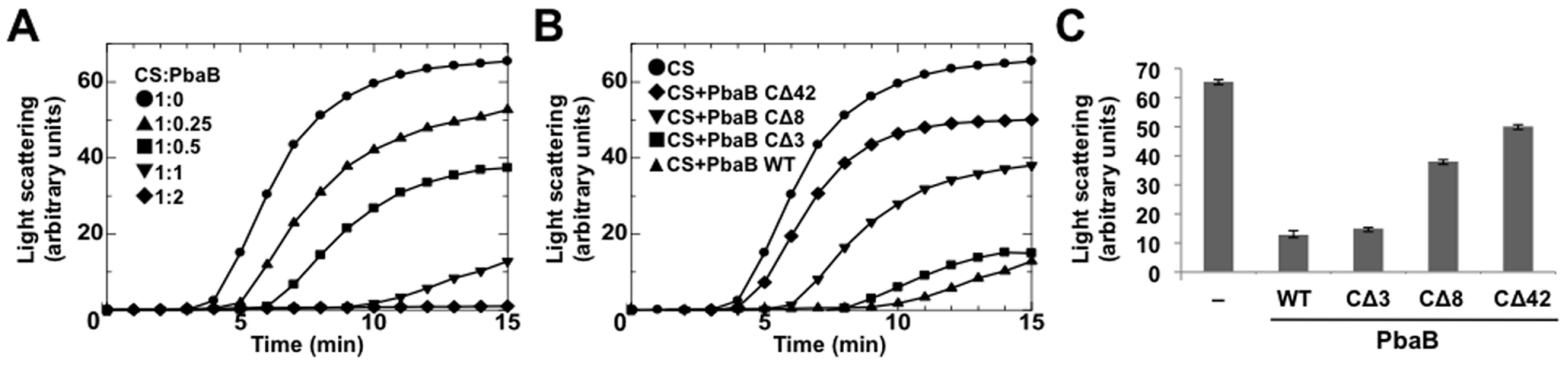
PbaB exerts a chaperone function through the C-terminal segments. (A) Aggregation of citrate synthase (CS, 50 nM) incubated at 50°C was monitored by measuring absorbance at 500 nm for 15 min; 1∶0 molar ratio (CS monomer:PbaB tetramer, circles), 1∶0.25 (triangles), 1∶0.5 (squares), 1∶1 (inverted triangles), and 1∶2 (diamonds). (B) Similar experiments were performed using the C-terminal deletion mutants of PbaB (1∶1 molar ratios); buffer (circles), wild-type PbaB (triangles), CΔ3 (squares), CΔ8 (inverted triangles), and CΔ42 (diamonds). (C) Similar results were obtained in three experiments. The graph indicates the values at 15 min. Error bars represent s.e.m.

Next, we addressed the issue how the active complex between the PbaB tetramer and 20S proteasome can recognize the unfolded proteins as substrates. In the proteasome activated with PAN, substrate proteins are captured by three tentacle-like coiled-coil pairs that project from the PAN ATPase face and are most distal from the 20S proteasome [30], [31], [32]. Subsequently, the substrates are unfolded by the action of ATPase and introduced into the proteasome catalytic channel through the central pore of the PAN hexamer [31]. It has been reported that the PAN hexamer exerts a chaperone function to suppress aggregation of denatured proteins [11] by interacting with them through the outer tentacle-like coiled-coil regions [30]. We examined whether the PbaB tetramer also has such molecular chaperone activity using citrate synthase (molecular mass, 85 kDa) as a model ligand. We found that aggregation of citrate synthase incubated at 50°C was significantly reduced in the presence of PbaB and almost completely suppressed in the presence of twofold molar excess of the PbaB tetramer (Figure 5A). We also confirmed that PbaB suppressed the aggregation of heat-denatured rhodanese (Figure S5). The aggregations were prevented in ATP-independent manners suggesting that the observed aggregate suppression was achieved simply through the binding of the denatured proteins to PbaB, which thereby holds them in a soluble state.

To identify the sites responsible for binding to the denatured proteins, we prepared PbaB mutants that lacked 3, 8, or 42 residues at the C-terminus and examined their chaperone activity. Based on circular dichroism (CD) data, we confirmed that these C-terminal deletion mutants maintained structural integrity (Figure S6). Truncation of the eight residues of the C-terminus significantly compromised chaperone activity, and deletion of the α6 helix markedly impaired the suppression of aggregation of citrate synthase (Figure 5B and 5C). These findings indicated that the tentacle-like C-terminal segments of the PbaB tetramer are critically involved in capturing unfolded proteins. These data showed that the substrate-binding site of PbaB is proximal to its proteasome-binding motif, indicating that proteasome activation and chaperone activity are both expressed by the C-terminal segments of the PbaB tetramer. Based on the electron tomography and biochemical data, we hypothesize that a subset of C-terminal segments function as gate openers to activate the 20S proteasome, and the remaining unanchored C-terminal segments are involved in capturing proteasomal substrates. It is possible that the PbaB tetramer can simultaneously bind the 20S proteasome and its substrate by the C-terminal dual interaction.

In summary, we revealed that PbaB acts as a novel proteasome activator in archaea. These findings provide insights into molecular evolution relationships between proteasome activators and assembly factors from a structural perspective.

While this manuscript was in review process, a paper appeared in press describing a structural study of yeast proteasome assembly chaperones Pba1/Pba2 [33]. Pba1 and Pba2 formed a heterodimer, although the overall structures were similar to that of PbaB.

## Materials and Methods

### Expression and purification of *P. furiosus* proteins

*P. furiosus* genomic DNA was purchased from RIKEN BioResource Center (Japan). For purification of *P. furiosus* PbaA and PbaB, the genes encoding full-length *pbaA* (PF0015, residues 1–242) and *pbaB* (PF1142, residues 1–280) were cloned into the *Nde*I and *Xho*I sites of the pET-28b vector (Novagen). The expressed proteins contained a His_6_-tag at the N-terminus. *E. coli* BL21(DE3) transformed with the plasmid was cultured in LB medium containing 15 μg/ml kanamycin and subsequently harvested after induction with 0.5 mM isopropyl β-D-thiogalactoside (IPTG, Wako Pure Chemical Industries) for 3 h at 37°C. Harvested cells were resuspended with buffer A [20 mM Tris-HCl (pH 7.5) and 150 mM NaCl] and lysed with sonication. The cell lysate was loaded on a Ni^2+^-charged chelating Sepharose column (GE Healthcare), and the bound protein fraction was extensively washed with buffer A supplemented with 20 mM imidazole. The His_6_-tagged protein was eluted with buffer A containing 500 mM imidazole. The eluted protein was then dialyzed against buffer B [50 mM Tris-HCl (pH 7.5)]. The protein was purified on a HiTrap Q HP anion exchange column (GE Healthcare) in buffer B containing 10 mM 2-mercaptoethanol and developed with a 0–1 M NaCl gradient. Further purification was performed using a HiLoad Superdex 200 column (GE Healthcare) in buffer B containing 10 mM 2-mercaptoethanol and 150 mM NaCl. For the co-expression experiments, *pbaA* and *pbaB* were cloned into the *Bam*HI/*Sal*I and *Nde*I/*Xho*I sites of pETDuet-1 vector (Novagen), respectively. The expressed PbaA possessed a His_6_-tag at the N-terminus, whereas PbaB was expressed as a non-tagged form. Deletion mutants of PbaB [CΔ3 (residues 1–277), CΔ8 (1–272), and CΔ42 (1–238)] were constructed using a PCR-based technique. The mutated proteins were expressed and purified using the same methods as employed for wild-type PbaB. For the preparation of non-tagged PbaB, the His_6_-tag was cleaved by thrombin protease treatment before HiTrap Q HP column chromatography.

For purification of the *P. furiosus* 20S proteasome, genes encoding *psmA* (PF1571; α subunit, residues 1–260) and *psmB1* (PF0159; β subunit 1, residues 1–196) were cloned into pET-28b (*Nco*I and *Bam*HI) and pET-21a (*Nde*I and *Bam*HI) vectors, respectively. We made two constructs for β1 subunit expression: C-terminally His_6_-tagged and non-tagged versions. For preparation of the 20S proteasome consisting of α subunits and two isoforms of β1 and β2 subunits, a gene encoding *psmB2* (PF1404; β subunit 2, residues 12–206) was cloned into pACYCDuet-1 (*Nco*I and *Bam*HI) vector. The recombinant β2 subunit protein was not modified with affinity tag and had a catalytically inactive mutation of T11A. For preparation of the 20S proteasome (αβ1 form), *E. coli* BL21(DE3) transformed with the plasmids were cultured in LB medium containing 50 μg/ml ampicillin and 15 μg/ml kanamycin and harvested after induction with 0.5 mM IPTG for 3 h at 37°C. Additional antibiotics, 20 μg/ml chloramphenicol was added for the expression of αβ1β2 form of 20S proteasome. The His_6_-tagged wild-type 20S proteasome (αβ1 form) and its inactive mutants of 20S proteasome [α + β1^His6/NΔ6/T7A^ (αβ1 form) and α + β1^His6/NΔ6/T7A^ + β2^NΔ10/T11A^ (αβ1β2 form)] were purified using the same procedure as that employed for the Pba proteins except for size exclusion chromatography in which a Sephacryl S-400 column (GE Healthcare) was used instead. For purification of the non-tagged 20S proteasome inactive mutants [α + β1^NΔ6/T7A^ (mature form) and α + β1^T7A^ (immature form)], the cell lysate was heat precipitated at 85°C for 20 min. Subsequent purification was performed using the same method employed for the His_6_-tagged form excluding immobilized metal ion affinity chromatography. Purification of the α subunit ring was performed using the same procedure as that for the non-tagged 20S proteasome mutants.

### Pull-down experiment

The His_6_-fused mature 20S proteasome [inactive mutants, α + β1^His6/NΔ6/T7A^ (αβ1 form) and α + β1^His6/NΔ6/T7A^ + β2^NΔ10/T11A^ (αβ1β2 form)] and non-tagged PbaA and PbaB were used in a pull-down assay. We carried out all pull-down experiments at physiological temperature of *P. furiosus* (85°C) to eliminate a non-specific interaction. For immobilization, 8 μg of the 60 nM His_6_-fused mature 20S proteasome mutants were applied on Ni^2+^-charged chelating Sepharose beads, which were subsequently incubated with 6 μg of 930 nM PbaA, PbaB, or their mixture for 30 min in 25 mM Tris-HCl (pH 7.5) and 150 mM NaCl. The beads were washed five times with the buffer containing 50 mM imidazole. Proteins bound to the beads were eluted by 500 mM imidazole and analyzed by SDS-PAGE. The gels were stained with Coomassie Brilliant Blue (CBB).

The co-expressing *E. coli* lysate containing His_6_-fused PbaA and non-tagged PbaB were used in a pull-down assay to examine PbaA-PbaB interaction. The cell lysate was prepared in a solution containing 25 mM Tris-HCl (pH 7.5) and 150 mM NaCl.

### Proteasome activation assay

All samples were dissolved in a solution containing 20 mM Tris-HCl (pH 7.5) and 150 mM NaCl. For the peptide substrate degradation assay, 10 μM fluorogenic nonapeptide substrate [LFP, mca-AKVYPYPME-dap(dnp)-amide] was mixed with 175 nM wild-type or mutated PbaB and 35 nM wild-type 20S proteasome (20S proteasome/PbaB molar ratio 1∶5) and incubated at 45°C for 15 min with a 1-min sampling interval. Hydrolysis of the LFP was monitored at λ_ex_ 330 nm and λ_em_ 398 nm. The buffer solution containing 1 mM ATP and 10 mM MgCl_2_ were used to assess the ATP dependency. For the protein substrate degradation assay, 18 μM human α-synuclein was mixed with 105 nM wild-type or mutated PbaB and 35 nM the wild-type 20S proteasome (20S proteasome/PbaB molar ratio 1∶3) and incubated at 95°C for ∼4 h with a 40-min sampling interval. Recombinant human α-synuclein was purified as previously described [34]. The enzymatic reaction was stopped upon boiling with SDS-PAGE loading buffer. Degradation of α-synuclein was monitored by SDS-PAGE. The gels were stained with CBB, and the intensity of the substrate band was measured using ImageJ software (http://rsbweb.nih.gov/ij/).

### Chaperone activity assay

Citrate synthase (50 nM) was incubated in 50 mM Tris-HCl (pH 7.5) in the presence or absence of His_6_-tagged wild-type or mutated PbaB for 15 min in a cuvette kept at 50°C with a 1-min sampling interval. To evaluate the aggregation level, light scattering was measured at 500 nm in a spectrophotometer and expressed in arbitrary units.

### Measurements of CD spectra

His_6_-tagged PbaB and the mutated proteins were dissolved in 10 mM sodium phosphate buffer (pH 7.4) containing 0.15 M NaCl. Measurements of CD spectra were performed in a 1-mm quartz cuvette at a room temperature using a spectropolarimeter (J-725, JASCO). After subtraction of the spectrum of the buffer alone, data were represented as mean residue ellipticities.

### SPR measurements

SPR measurements were performed at 25°C using a Biacore T100 system (Biacore) equipped with a Sensor Chip NTA (Biacore). The His_6_-tagged PbaB was immobilized on the flow cell. Non-tagged mature or immature 20S proteasomes or an α subunit ring at concentrations of 18.8–300 nM, 18.5–296 nM or 15.4–246 nM, respectively, in a running buffer [10 mM HEPES (pH 7.5), 150 mM NaCl, and 0.05% Tween20] were injected over the flow cells at a flow rate of 10 μl/min.

### Analytical ultracentrifugation

Using a Proteomelab XL-I Analytical Ultracentrifuge (Beckman – Coulter), a sedimentation velocity (SV) experiment was performed using 20 μM His_6_-tagged PbaA or PbaB in 150 mM sodium phosphate (pH 7.5), containing 100 mM NaCl. Runs were performed at 38,000 rpm and 20°C using 12-mm charcoal epon double sector centerpieces and a four-hole An60 Ti analytical rotor that was equilibrated to the same temperature. The sedimentating boundary was monitored using absorbance detection optics at 237 nm for PbaA, and PbaB, respectively. The radial increment was 0.003 cm and at least 150 scans were obtained between 5.9 cm and 7.25 cm from the center of the rotation axis. The partial specific volumes for PbaA and PbaB were calculated as 0.7506 ml•g^−1^ and 0.7424 ml•g^−1^, respectively using Sednterp1.09. All SV raw data were analyzed using the continuous *C*(s) distribution model provided by the software program SEDFIT11.71 [35].

### Crystallization, X-ray data collection, and structure determination

Non-tagged PbaB was used for crystallization. PbaB was dissolved at 7 mg/mL in 50 mM Tris-HCl (pH 7.5), and the crystal was obtained in a buffer containing 10% PEG10000, 100 mM Bis-Tris (pH 5.0), 50 mM sodium acetate, and 5 mM NaAuCl_4_ on incubation at 20°C for 1 week. All crystals were cryoprotected with crystallization mother liquor supplemented with 20% ethylene glycol. The native and anomalous datasets were collected at wavelengths of 0.90000 Å and 1.03514 Å, respectively (BL44XU at SPring-8). The crystals of PbaB belonged to space group *C*222_1_ and diffracted up to a resolution of 2.20 Å. All diffraction data were processed using HKL2000 [36]. The crystal parameters of PbaB are shown in Table 1.

The crystal structure of PbaB was solved by the SAD method using modules of the Phenix suite [37], with anomalous scattering substructure searches, density modification, and automated model building from the AutoSol wizard. Using the 2.20 Å native dataset, further automated model building and manual model fitting to electron density maps were performed using ARP/wARP [38] and COOT [39], respectively. The refinement procedure was performed using REFMAC5 [40]. The final model of PbaB contained residues 14–257 (molecule A), 14–257 (B), 14–261 (C), and 12–259 (D). The stereochemical quality of the final model was assessed using RAMPAGE [41]. The refinement statistics of PbaB are summarized in Table 1. The molecular graphics were prepared using PyMOL (http://www.pymol.org/).

### Electron tomography and subtomogram averaging

Electron tomography and subtomogram averaging were performed as previously described [42]. The His_6_-tagged mature 20S proteasome (inactive mutant, α + β^His6/NΔ6/T7A^) and His_6_-tagged PbaB were mixed at a molar ratio of 1∶5 (final concentration, 0.5 mg/ml) for 2 h at room temperature. EM grids were prepared according to the conventional negative staining protocol [43]. Samples were imaged at room temperature using a JEOL JEM 2200FS electron microscope equipped with a field emission gun operating at an acceleration voltage of 200 kV. Images were taken at a detector magnification of 61,425× of a defocus value of 1.5 μm and a zero-loss energy slit of 50 eV on Tietz F416 4kx4k CCD camera using a low dose procedure. Tilt series were collected at a single axis angular increment of 2° with ±70° specimen tilt. Pixel size was 2.44 Å/pixel on specimen position. Tomographic reconstruction was performed with IMOD software using particles as a fiducial marker [44]. 159 subtomograms of the PbaB-20S proteasome complexes were extracted from two reconstructed tomograms with a cube of 150×150×150 pixels. The alignment was done as follows using EMAN software [45]. There were two types of subtomograms. Some were singly capped with one PbaB tetramer on a side of the α subunit ring; others were double capped with two PbaB tetramers on both sides of the α subunit ring. Therefore, subtomograms of the complex were aligned with the atomic model of the 20S proteasome (PDB ID:3MI0) after centering the volumes and removing the densities of the PbaB tetramer by a tight spherical mask. Then, the density of PbaB was oriented to the same side of that of the 20S proteasome in the volume, and the volumes were rotationally aligned around the seven-fold axis for averaging. 42 of 91 and 9 of 21 subtomograms including tiled and parallel caps of the PbaB tetramer, respectively, were finally selected in the singly capped complexes for averaging. The atomic models of the 20S proteasome and the PbaB tetramer were fit into the individual densities using UCSF Chimera [46].

## Supporting Information

Figure S1 **Surface plasmon resonance analyses between PbaB and proteasome derivatives.** (A) Inactive mature 20S proteasome (α + β1^NΔ6/T7A^), (B) immature 20S proteasome (α + β1^T7A^), or (C) α subunit ring was injected into a PbaB-immobilized biosensor chip at five concentrations, i.e., serial 2-fold dilutions of proteasome derivatives staring from 300 nM (A) 296 nM (B), 246 nM (C). The dissociation constants of the α subunit ring and inactive immature or mature 20S proteasomes were estimated to be 3.3±0.6×10^−7^ and 2.1±0.3×10^−8^ or 7.8±0.6×1^−9^ M, respectively. (D) Comparison of sensorgrams of inactive mature 20S proteasome injection (300 nM) into biosensor chips with immobilized PbaB in wild-type or CΔ3-mutated form.
(TIF)

Figure S2 **SDS-PAGE of 20S proteasome (αβ1 form) and PbaB.** The 20S proteasome and PbaB were incubated at 95°C for 4 h.
(TIF)

Figure S3 **Analytical ultracentrifugation of PbaA (red) and PbaB (blue).** Sedimentation coefficient distribution of the Pba proteins obtained from analysis of sedimentation velocity experiments. These data demonstrated that PbaA and PbaB form a homopentamer and homotetramer, respectively.
(TIF)

Figure S4 **Crystal structures of PbaB (A and C) and a PbaA homolog Ta1441 (B and D).** β-strands and α-helices are highlighted in magenta and yellow, respectively. In A and B, protomers of Pba homologs are represented. (B) Ribbon diagram of *Thermoplasma acidophilum* Ta1441 is originated from the crystal structure (PDB: 3GAA). PbaB (C) and Ta1441 (D) form a homotetramer and homopentamer, respectively.
(TIF)

Figure S5 **PbaB prevented thermal aggregation of rhodanese.** Rhodanese from the bovine liver (50 nM) was incubated at 50°C and its aggregation was monitored by measuring optical density at 500 nm for 15 min; 1∶0 molar ratio (Rhodanese:PbaB tetramer, circles), 1∶0.25 (triangles), 1∶0.5 (squares), 1∶1 (invert triangles), and 1∶2 (diamonds).
(TIF)

Figure S6 **CD spectra of wild-type and mutated PbaB.** Wild type (black); CΔ3 (green); CΔ8 (blue); CΔ42 (yellow).
(TIF)
