# Supplementary figures and images for: An Archaeal Homolog of Proteasome Assembly Factor Functions as a Proteasome Activator
Source: PLoS One. 2013 Mar 21;8(3):e60294. doi: 10.1371/journal.pone.0060294 (PMC3605417; doi:10.1371/journal.pone.0060294)

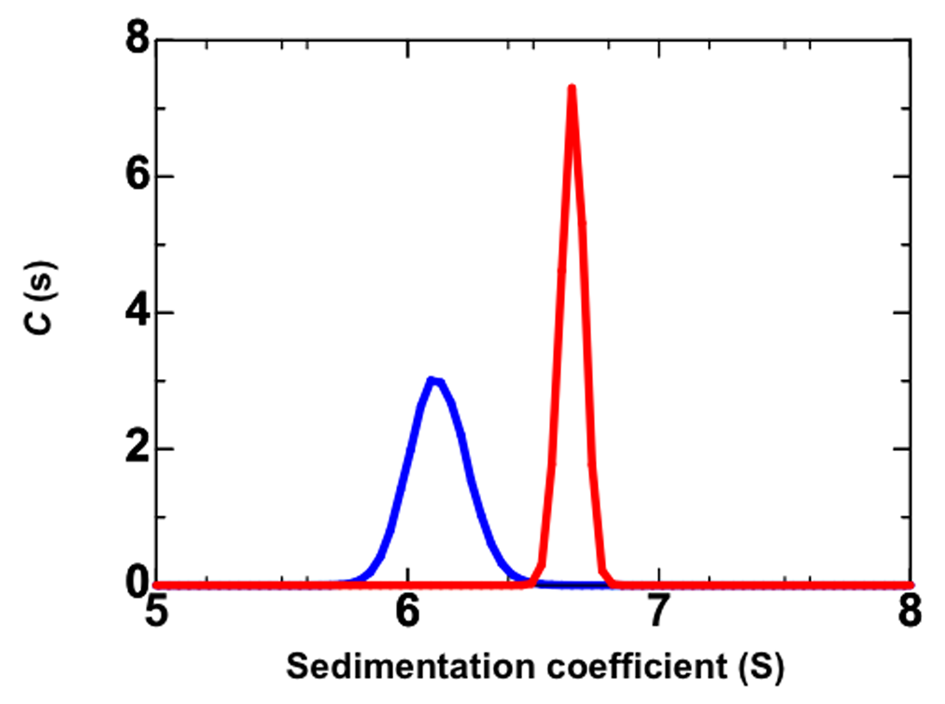

Supplement: Figure S3 — Analytical ultracentrifugation of PbaA (red) and PbaB (blue). Sedimentation coefficient distribution of the Pba proteins obtained from analysis of sedimentation velocity experiments. These data demonstrated that PbaA and PbaB form a homopentamer and homotetramer, respectively. (TIF) [file pone.0060294.s003.tif]

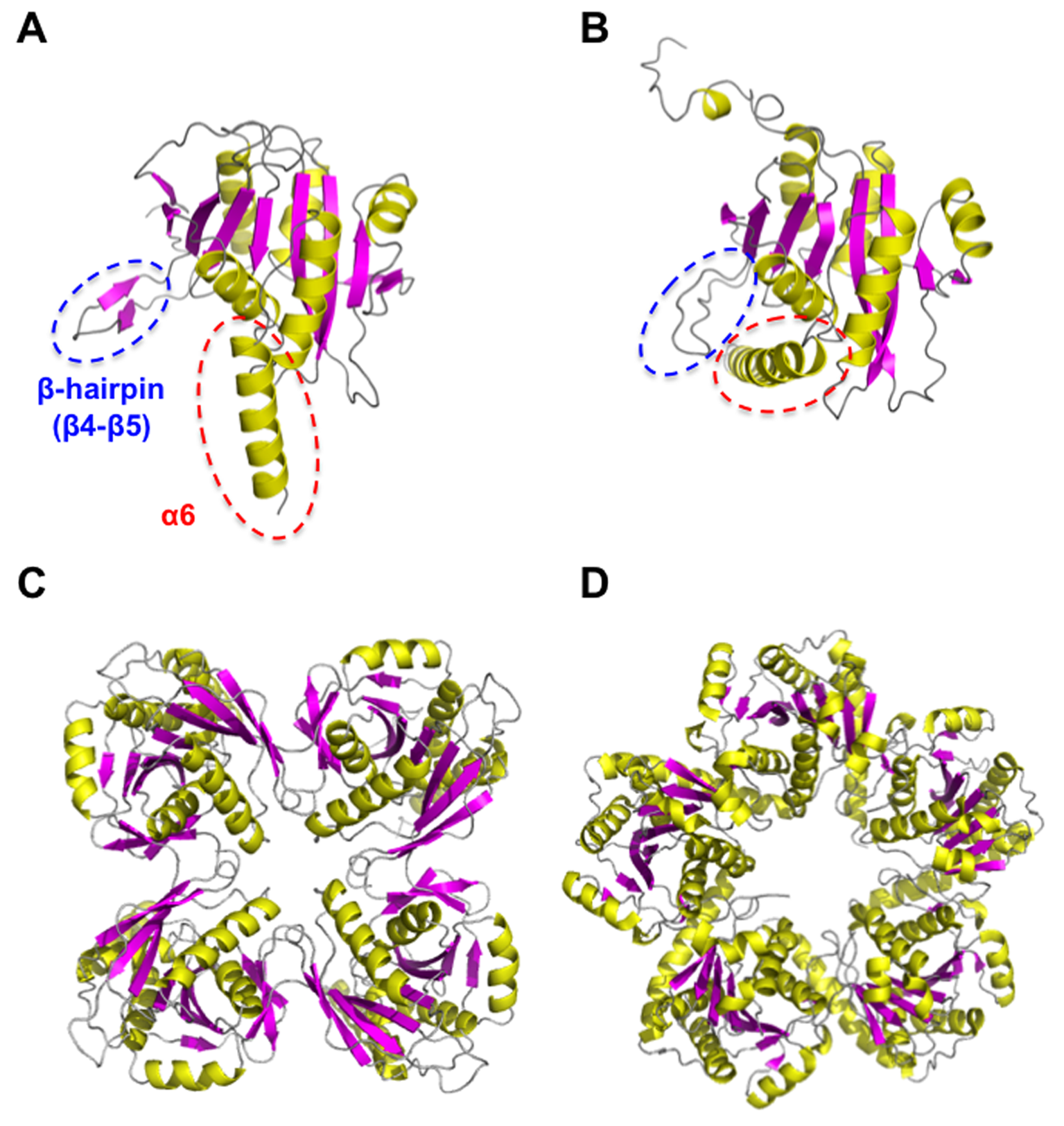

Supplement: Figure S4 — Crystal structures of PbaB (A and C) and a PbaA homolog Ta1441 (B and D). β-strands and α-helices are highlighted in magenta and yellow, respectively. In A and B, protomers of Pba homologs are represented. (B) Ribbon diagram of Thermoplasma acidophilum Ta1441 is originated from the crystal structure (PDB: 3GAA). PbaB (C) and Ta1441 (D) form a homotetramer and homopentamer, respectively. (TIF) [file pone.0060294.s004.tif]

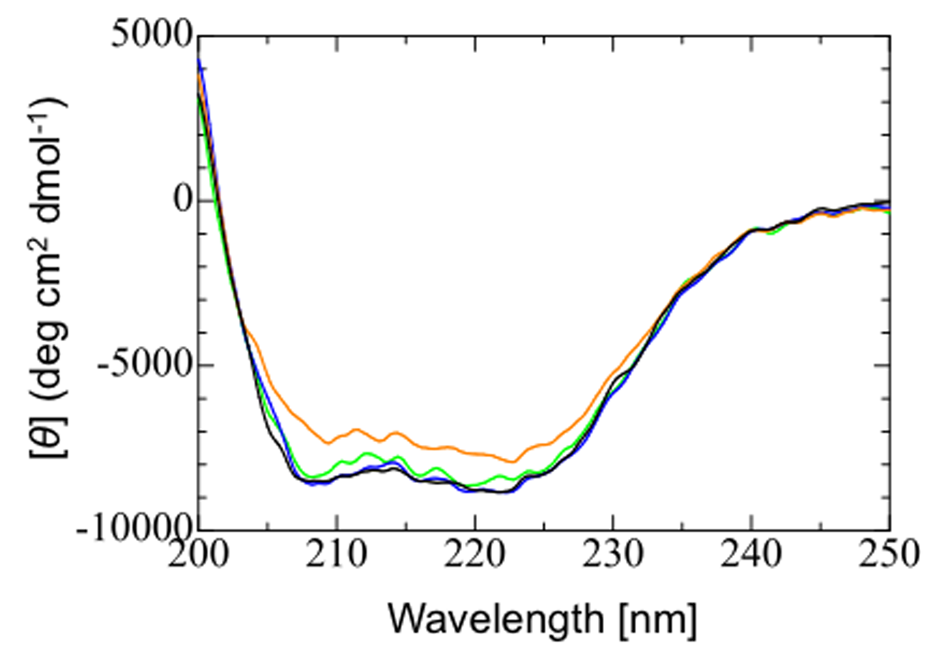

Supplement: Figure S6 — CD spectra of wild-type and mutated PbaB. Wild type (black); CΔ3 (green); CΔ8 (blue); CΔ42 (yellow). (TIF) [file pone.0060294.s006.tif]
